# Identification of an immune-responsive mesolimbocortical serotonergic system: Potential role in regulation of emotional behavior

**DOI:** 10.1016/j.neuroscience.2007.01.067

**Published:** 2007-05-11

**Authors:** C.A. Lowry, J.H. Hollis, A. de Vries, B. Pan, L.R. Brunet, J.R.F. Hunt, J.F.R. Paton, E. van Kampen, D.M. Knight, A.K. Evans, G.A.W. Rook, S.L. Lightman

**Affiliations:** a Henry Wellcome Laboratories for Integrative Neuroscience and Endocrinology, University of Bristol, Dorothy Hodgkin Building, Bristol BS1 3NY, UK; b Centre for Infectious Diseases and International Health, Windeyer Institute of Medical Sciences, University College London, W1T 4JF, UK; c Department of Physiology, Bristol Heart Institute, University of Bristol, School of Medical Sciences, Bristol, BS8 1TD, UK

**Keywords:** depression, hippocampus, prefrontal cortex, raphe, 5-HT, vagus, ANOVA, analysis of variance, AP, area postrema, c-Fos-ir, c-Fos-like-immunoreactive, DR, dorsal raphe nucleus, DRC, dorsal raphe nucleus, caudal part, DRI, dorsal raphe nucleus, interfascicular part, ECG, electrocardiogram, EDTA, ethylenediaminetetraacetic acid, EMG, electromyogram, HPLC, high pressure liquid chromatography, IL-6, interleukin-6, IL-10, interleukin-10, i.t., intratracheal, LPS, lipopolysaccharide, LSD, least significant difference, mlf, medial longitudinal fasciculus, *M. vaccae*, *Mycobacterium vaccae*, Mv-NC, *Mycobacterium vaccae* antigen, *M. vaccae* coupled to nitrocellulose beads, NC, nitrocellulose beads, nTS, nucleus of the solitary tract, OVA, ovalbumin, OVA-NC, ovalbumin coupled to nitrocellulose beads, PBG, phenylbiguanide, PBS, phosphate-buffered saline, PBST, phosphate-buffered saline containing 0.3% Triton X-100, RMg, raphe magnus, ROb, raphe obscurus, S.E.M., standard error of the mean, SolDL, dorsolateral part of the nucleus of the solitary tract, TGF-β, transforming growth factor-β, Th1, T helper cell 1, Th2, T helper cell 2, TNF-α, tumor necrosis factor-α, Treg, T regulatory cell, 5-HIAA, 5-hydroxyindoleacetic acid, 5-HT, serotonin

## Abstract

Peripheral immune activation can have profound physiological and behavioral effects including induction of fever and sickness behavior. One mechanism through which immune activation or immunomodulation may affect physiology and behavior is via actions on brainstem neuromodulatory systems, such as serotonergic systems. We have found that peripheral immune activation with antigens derived from the nonpathogenic, saprophytic bacterium, *Mycobacterium vaccae*, activated a specific subset of serotonergic neurons in the interfascicular part of the dorsal raphe nucleus (DRI) of mice, as measured by quantification of c-Fos expression following intratracheal (12 h) or s.c. (6 h) administration of heat-killed, ultrasonically disrupted *M. vaccae*, or heat-killed, intact *M. vaccae*, respectively. These effects were apparent after immune activation by *M. vaccae* or its components but not by ovalbumin, which induces a qualitatively different immune response. The effects of immune activation were associated with increases in serotonin metabolism within the ventromedial prefrontal cortex, consistent with an effect of immune activation on mesolimbocortical serotonergic systems. The effects of *M. vaccae* administration on serotonergic systems were temporally associated with reductions in immobility in the forced swim test, consistent with the hypothesis that the stimulation of mesolimbocortical serotonergic systems by peripheral immune activation alters stress-related emotional behavior. These findings suggest that the immune-responsive subpopulation of serotonergic neurons in the DRI is likely to play an important role in the neural mechanisms underlying regulation of the physiological and pathophysiological responses to both acute and chronic immune activation, including regulation of mood during health and disease states. Together with previous studies, these findings also raise the possibility that immune stimulation activates a functionally and anatomically distinct subset of serotonergic neurons, different from the subset of serotonergic neurons activated by anxiogenic stimuli or uncontrollable stressors. Consequently, selective activation of specific subsets of serotonergic neurons may have distinct behavioral outcomes.

Chronic immune-related disease is associated with major depression and suicidal ideation (Chang et al., 2001; Chaney et al., 1999; Druss and Pincus, 2000; Hurwitz and Morgenstern, 1999). It is unclear if this association is secondary to a decreased quality of life (Chang et al., 2001), shared genetic vulnerability to chronic immune dysfunction and major depression (Wamboldt et al., 2000), or if it reflects a cause and effect relationship (Capuron and Miller, 2004; Wamboldt et al., 2000). Chronic immune activation with interferon α or interleukin-2 (IL-2) induces depressive symptoms in human patients and treatment with antidepressant drugs acting on serotonergic systems can prevent the onset of depressive symptoms (Capuron and Miller, 2004; Capuron et al., 2004), suggesting that serotonergic systems may play an important role in the relationship between immune function and affective state. A critical issue for understanding these relationships is to determine the effects of immune activation on neural systems regulating mood, particularly serotonergic systems.

Serotonergic systems are important modulators of behavioral arousal, motor activity, and mood (Jacobs and Azmitia, 1992; McAllister-Williams et al., 1998). The majority of serotonergic neurons, referred to as Type I serotonergic neurons, display a high spontaneous firing rate during active waking states and a progressively lower spontaneous firing rate during inactive states, with a complete cessation of activity during rapid eye movement (REM) sleep (Rasmussen et al., 1984). However, an interesting paradox occurs following acute immune activation; behavioral activity dramatically *decreases* while serotonergic activity *increases*, particularly in limbic brain regions associated with mood regulation (Linthorst et al., 1995). One potential explanation for these findings is that immune activation increases the activity of a subpopulation of serotonergic neurons that is not directly associated with the level of behavioral arousal. Electrophysiological studies have identified a small subpopulation of serotonergic neurons, referred to as Type II serotonergic neurons, that have unique electrophysiological properties and behavioral correlates (Rasmussen et al., 1984; Sakai and Crochet, 2001). Type II serotonergic neurons display firing rates that are independent of the level of behavioral arousal; these neurons are restricted to a highly confined region of the brainstem raphe complex at the caudal interface of the dorsal and median raphe nuclei, between the medial longitudinal fasciculi (mlf), a region defined as the interfascicular part of the dorsal raphe nucleus (DRI) in mice, rats, and primates (Azmitia and Gannon, 1986; Paxinos and Watson, 1998; Paxinos and Franklin, 2001), that gives rise to projections to limbic forebrain regions (Azmitia, 1981; Porrino and Goldman-Rakic, 1982; Van Bockstaele et al., 1993). It is not yet clear if this region contains exclusively Type II serotonergic neurons or alternatively contains both Type I and Type II serotonergic neurons.

To investigate the effects of immunomodulation and immune activation on serotonergic neurons we elicited a localized bronchopulmonary T helper cell 1 (Th1)/T regulatory cell (Treg) response by challenging *Mycobacterium vaccae* (*M. vaccae*) preimmunized mice with *Mycobacterium vaccae* antigens (Mv-NC), or a T helper cell 2 (Th2) response by challenging ovalbumin (OVA)/alum preimmunized mice with OVA, then conducted neuroanatomical mapping of immediate-early gene expression and measured serotonin (5-HT) and 5-HT metabolite concentrations in forebrain structures receiving mesolimbocortical serotonergic input. In addition, to determine the effects of immune activation with *M. vaccae* on stress-related emotional behavior, we measured behavioral responses to *M. vaccae* administration in the forced swim test.

## Experimental procedures

### Animals

Adult male specific pathogen free (SPF) BALB/c mice (6–8 weeks old, 21–25 g) were group housed at 22 °C on a 12-h light/dark cycle (lights on at 7:00 A.M.; University College London, experiments 1, 2, 4) or on a 14-h L:10-h D light/dark cycle (lights on at 5:00 A.M.; University of Bristol, experiments 3, 5, 6). All animal experiments were performed in accordance with the UK Animals (Scientific Procedures) Act, 1986 under protocols approved by the UK Home Office and the Institutional Animal Care and Use Committee of University College London or the Ethical Review Group at the University of Bristol. In addition, all studies were consistent with the U.S. National Institutes of Health Guide for the Care and Use of Laboratory Animals (NIH Publication No. 85-23) and were covered by Animal Welfare Assurance #A5057-01. All efforts were made to minimize the number of animals used and their suffering.

### Preimmunization

Unless specified otherwise, all mice were preimmunized s.c. with 0.1 mg whole heat-killed *M. vaccae* (10 mg/ml suspension of heat-killed *M. vaccae* (SRP 299) diluted to 1 mg/ml) in 100 μl sterile saline on day −28 and day −14. Sterile heat-killed suspension of *M. vaccae* was provided by SR Pharma (London, UK). The material was identical to material that has been tested in phase II clinical trials in cancer (O’Brien et al., 2000, 2004) and inflammatory disorders (Dalbeth et al., 2004) and has resulted in unexpected improvements in quality of life scores (O’Brien et al., 2004). The dose used in these experiments (0.1 mg) was 1/10 of the dose used in human studies (1 mg) (O’Brien et al., 2000, 2004; Dalbeth et al., 2004) and identical to the dose used in previous studies in mice (Zuany-Amorim et al., 2002).

### Murine model of bronchopulmonary immune activation

Initial experiments sought to expand preliminary findings on endocrine effects of tuberculosis (Rook et al., 1997), an infectious disease caused by the bacterium *Mycobacterium tuberculosis*, and therefore we used methods designed to produce a localized immune activation in the airways with the related, non-pathogenic bacterium, *M. vaccae*. As we were interested in the effects of localized peripheral immune activation on the CNS, we used nitrocellulose beads (NC) in an attempt to prevent dispersal of antigens into the systemic circulation and therefore to localize the immune activation to the bronchopulmonary system (Abou-Zeid et al., 1987). Furthermore, we compared immune stimuli designed to elicit either a Th1-dominant response (*M. vaccae*, as an experimental model of tuberculosis) or a Th2-dominant response (OVA, as an experimental model of asthma). Administration of *M. vaccae* in preimmunized mice results in rapid release of mediators from memory lymphocytes of the Th1 (Skinner et al., 2001; Abou-Zeid et al., 1997; Hernández-Pando et al., 1997) and Treg subsets (Zuany-Amorim et al., 2002), whereas administration of OVA in OVA/alum preimmunized mice results in rapid release of mediators from memory lymphocytes of the Th2 subset (Zuany-Amorim et al., 2002). *M. vaccae* administration has also been shown to facilitate the development of Treg cells that can downregulate Th2 immune responses via production of interleukin-10 (IL-10) and transforming growth factor-β (TGF-β) (Zuany-Amorim et al., 2002). For administration of *M. vaccae* into the airways, mice were anesthetized with 1:1 Hypnorm/Hypnovel mixture (i.p., Hypnorm: 0.32 mg/kg fentanyl citrate and 10 mg/kg fluanisone; Janssen Pharmaceuticals Ltd., Oxford, UK; Hypnovel: 10 mg/2 ml midazolam, Hoffmann-La Roche Ltd., Basel, Switzerland) diluted 1:1 with water. Following exposure of the ventral surface of the trachea, intratracheal (i.t.) injections of sonicated, heat-killed *M. vaccae* (6 μg/50 μl) coupled to nitrocellulose beads (Mv-NC) or nitrocellulose beads alone (NC), were given using a sterile 23 ga. needle. Sonicated, heat-killed *M. vaccae* was coupled to NC using methods described previously (Abou-Zeid et al., 1987). Following injection, anesthetized mice were maintained in a vertical position for 2–3 min to facilitate flow of injectate into the upper airways. For methods for pre-immunization and immune challenge with OVA, see experiments 1 and 2 (below).

### Details of individual experiments

#### Experiment 1

To determine the effects of i.t. injections of Mv-NC on c-Fos expression in DRI serotonergic neurons, as well as on serotonergic neurons in the caudal dorsal raphe nucleus (DRC), mice (*n*=4–6) were preimmunized on days −28 and −14 with whole heat-killed *M. vaccae* (0.1 mg s.c.) suspended in saline and injected i.t. on day 0 with Mv-NC (6 μg in 50 μl) or NC alone. Following treatment (12 h or 3, 6, 10, or 17 days), mice were killed by overdose of halothane and blood samples were collected by cardiac puncture. Blood samples were mixed with 50 μl of 0.5 M EDTA, and kept on ice until centrifugation at 10,000 r.p.m. for 10 min; plasma was stored at −20 °C until used for plasma cytokine measurements. For semi-quantitative measurement of pulmonary IL-1β, tumor necrosis factor-α (TNF-α), and interleukin-6 (IL-6) mRNA expression (for detailed methods see below), additional mice (*n*=5–6) were preimmunized on days −28 and −14 with 50 μg OVA on 2 mg alum (i.p.), suspended in saline and injected i.t. on day 0 with ovalbumin coupled to nitrocellulose beads (OVA-NC) (5 μg in 50 μl) or NC alone. For measurement of pulmonary cytokine mRNA expression in mice injected with NC alone, measurements were made at the 12 h time point only. Following overdose of mice with anesthetic, collection of blood using cardiac puncture, and removal of lung tissues, brains were fixed by transcardial perfusion of ice-cold 0.05 M phosphate buffered saline (PBS; pH 7.4) followed by ice-cold 4% paraformaldehyde in 0.1 M sodium phosphate buffer (PB; pH 7.4) in preparation for immunohistochemistry.

#### Experiment 2

To compare the effects of qualitatively different types of bronchopulmonary immune activation on c-Fos expression in DRI and DRC serotonergic neurons, mice were preimmunized on days −28 and −14 with either 1) whole heat-killed *M. vaccae* (0.1 mg s.c.) or 2) 50 μg OVA on 2 mg alum (i.p.), suspended in saline. Mv-NC or OVA challenge was conducted on day 0. Anesthetized mice preimmunized with *M. vaccae* were given i.t. injections of Mv-NC (6 μg in 50 μl) or NC alone (*n*=8); anesthetized mice preimmunized with OVA/alum were given i.t. injections of OVA-NC (5 μg in 50 μl) or NC alone (*n*=8). After 12 h mice were killed by overdose of halothane and brains were fixed by transcardial perfusion, as described above for experiment 1, in preparation for immunohistochemistry.

#### Experiment 3

To determine if pharmacological activation of afferent vagal fibers was sufficient to increase c-Fos expression in DRI or DRC serotonergic neurons, we injected the 5-HT_3_ receptor agonist phenylbiguanide (PBG) directly into the right atrium of mice, a technique that has been used previously to selectively activate cardiopulmonary 5-HT_3_ receptors in mice (Coleridge and Coleridge, 1984; Paton and Butcher, 1998). We used a modest dose of PBG (1–1.5 μg) in an attempt to selectively activate 5-HT_3_ receptors within the cardiovascular and pulmonary vascular beds. Although we cannot rule it out completely we believe it is unlikely that the modest doses of PBG used had direct effects on the CNS by passing across the blood–brain barrier or by actions at circumventricular organs that lack a blood–brain barrier. The dose used was relatively low (as used previously in mice; Paton and Butcher, 1998) and would become quickly diluted soon after activation of cardiopulmonary receptors. Moreover, in the mice used in the present study we did not find a PBG-evoked change in c-Fos expression in the AP (published elsewhere; see de Vries et al., 2005), a circumventricular organ that is known to have a high density of 5-HT_3_ receptors (Kilpatrick et al., 1989). Methods for intra-atrial injections of the 5-HT_3_ receptor agonist PBG have been described previously (de Vries et al., 2005). Briefly, adult male BALB/c mice (28–33 g; *n*=5) were used. Mice were lightly anesthetized by inhalation of halothane (Halothane:Fluothane, Sigma, UK). After weighing the lightly anesthetized mice, a deep anesthesia was induced using i.p. injection of a 1:1 Hypnorm/Hypnovel mixture. Subsequently, a cannula was inserted into the right atrium via the right jugular vein; the position of the cannula was verified postmortem. Stainless steel pins were placed s.c. in the chest wall to record the electrocardiogram (ECG); heart rate was derived from the ECG recording. The stainless steel pins also were used to generate an electromyogram (EMG) in order to monitor activity of respiratory muscles. The ECG and EMG were recorded using Neurolog amplifiers and filters (Digitimer, Welwyn Garden City, Hertfordshire, UK) and collected via an A:D interface (1401micro, Cambridge Electronic Design, Cambridge, UK) on a computer using Spike2 software (Cambridge Electronic Design). Immediately after finishing the surgery, a supra-threshold dose of PBG (Sigma-Aldrich, Gillingham, UK) in 0.9% sterile saline was established for each mouse by determining the dose required to induce an immediate decrease in heart rate and respiratory frequency. Control mice received a right atrial injection of 0.9% sterile saline. The temperature of the mice was monitored and maintained at 37 °C. To keep background c-Fos expression to a minimum as a result of the surgery, mice were maintained under anesthesia for 2 h prior to any treatment. For PBG treatment, mice received right atrial injections of a suprathreshold dose of PBG (1–1.5 μg, in 10–15 μl saline). This was repeated five times in total with an interval of 8–10 min between each injection. For control mice, each received five saline injections (10–15 μl) also at 8–10 min intervals. Mice were killed 2 h after the first of five PBG or saline injections by an overdose of anesthetic (sodium pentobarbital) injected through the jugular cannula. Brains were fixed by transcardial perfusion, as described above for experiment 1, in preparation for immunohistochemistry.

#### Experiment 4

To determine if the effects of Mv-NC on DRI serotonergic neurons were dependent on the site of peripheral immune activation, and to determine the time course of the responses in the DRI, mice were preimmunized on days −28 and −14 with whole, heat-killed *M. vaccae* as described above, then treated with either i.t. or s.c. injections of Mv-NC or *M. vaccae* itself, respectively. For i.t. injections, preimmunized mice were injected with either Mv-NC (6 μg/50 μl) or NC alone. For s.c. injections, because localizing the antigen to a specific organ system was not a primary objective, and to approximate the intradermal route of *M. vaccae* administration used in clinical studies, preimmunized mice were injected with either 0.1 mg/100 μl whole heat-killed *M. vaccae* (10 mg/ml suspension of heat-killed *M. vaccae* (SRP 299) diluted to 1 mg/ml) or saline alone. Mice were killed 2, 6, 12, or 24 h following treatment (*n*=6). Mice were killed by overdose of halothane and brains were fixed by transcardial perfusion, as described above for experiment 1, in preparation for immunohistochemistry.

#### Experiment 5

To determine if the effects of *M. vaccae* on DRI serotonergic neurons were associated with increased 5-HT metabolism in limbic forebrain structures, we investigated the effects of s.c. *M. vaccae* on tissue concentrations of l-tryptophan, 5-HT, and the 5-HT metabolite 5-hydroxyindoleacetic acid (5-HIAA) in the prelimbic cortex, infralimbic cortex, and the CA1 region of the hippocampus. We also measured total plasma concentrations of tryptophan to determine if treatment with *M. vaccae* altered peripheral tryptophan metabolism. On day 0, starting at 8:00 P.M., time-matched pairs of *M. vaccae*–preimmunized BALB/c mice received s.c. injections of either 0.1 mg/100 μl whole, heat-killed *M. vaccae* (10 mg/ml suspension of heat-killed *M. vaccae* (SRP 299) diluted to 1 mg/ml) or saline alone (*n*=9). After 12 h mice were killed by rapid decapitation and brains were removed and rapidly frozen on dry ice in preparation for sectioning, microdissection, and high pressure liquid chromatography (HPLC) with electrochemical detection for measurement of indoles. Blood samples were mixed with 50 μl of 0.5 M EDTA, and kept on ice until centrifugation at 10,000 r.p.m. for 10 min; plasma was stored at −20 °C until used for HPLC with electrochemical detection for measurement of tryptophan concentrations.

#### Experiment 6

To determine if the effects of *M. vaccae* on c-Fos expression in DRI serotonergic neurons, as well as 5-HT metabolism in limbic forebrain sites, was associated with changes in emotional behavior, we treated preimmunized and non-preimmunized mice with s.c. injections of *M. vaccae* and measured behavioral responses in the forced swim test, 12 and 36 h later. On days −28 and −14, BALB/c mice were preimmunized with *M. vaccae* as described above, or were given saline vehicle injections (s.c.). On day 0 at 8:00 P.M. time-matched groups of four mice (one from each treatment group) received s.c. injections of 0.1 mg/100 μl whole, heat-killed *M. vaccae* (10 mg/ml suspension of heat-killed *M. vaccae* (SRP 299) diluted to 1 mg/ml) or saline alone (*n*=8) in 15 min intervals until 10:00 P.M. Starting 12 h after the first injections (i.e. in 15 min intervals between the hours of 8:00 A.M. and 10:00 A.M. such that the post-injection interval was 12 h for all mice) the time-matched groups of four mice were subjected to the Porsolt forced swim test (Borsini, 1995). For details, see below.

### General methods

#### Measurement of plasma cytokines

Whole blood was centrifuged and plasma was stored at −20 °C until ELISA using ELISA kits (R & D Systems, Abingdon, Oxford, UK). Sensitivities of assays for IL-1β, TNF-α, and IL-6 were 7.8, 23.4 and 15.6 pg/ml, respectively.

#### Semi-quantitative measurement of pulmonary cytokine mRNA expression

Semi-quantitative RT-PCR was used to measure IL-1β, TNF-α, and IL-6 mRNA expression in the lungs. Lung tissue from each mouse was removed, frozen in liquid nitrogen, and homogenized in 1.5 ml Trizol (10 min). The homogenate was then transferred to an Eppendorf tube and was passed several times (for approximately 1 min) through a fine gauge needle. RNA from homogenized tissue was then extracted as recommended by the manufacturer (Gibco BRL, Paisley, UK). RNA was reverse transcribed using Superscript™ RT RNase H Reverse Transcriptase (Gibco BRL) followed by PCR using *Taq* polymerase (Gibco BRL) and appropriate primers for IL-1β, TNF-α, IL-6, and β-actin: IL-1β, 5′: 5′-ATGGCAACTGTTCCTGAACTCAACT-3′; 3′: 5′-CAGGACAGGTATAGATTCTTTCCTTT-3′, 563 bp; TNFα, 5′: 5′-ATGAGCACAGAAAGCATGATCCGC-3′; 3′: 5′-CCAAAGTAGACCTGCCCGGACTC-3′, 692 bp; IL-6, 5′: 5′-ATGAAGTTCCTCTCTGCAAGAGACT-3′; 3′: 5′-CACTAGGTTTGCCGAGTAGATCTC-3′, 638 bp; β-actin, 5′: 5′-GTGGGCCGCTCTAGGCACCAA-3′; 3′: 5′-CTCTTTGATGTCACGCACGATTTC-3′, 540 bp. PCR products were visualized on agarose gels; relative band densities were measured from scanned images (Fuji Image Gauge 3.01, Raytek Scientific, Sheffield, UK). Semi-quantitative measurements were based on comparisons of band densities with band densities of β-actin.

#### Immunohistochemistry

Every sixth section (30 μm) of the midbrain, pons, and medulla was used for double immunostaining using an antiserum directed against the protein product of the immediate-early gene, *c*-*fos* (rabbit anti-c-Fos polyclonal antiserum, PC-38 (Ab-5), 1:12,000; Merck Biosciences, Nottingham UK), followed by immunostaining using (midbrain and pons only) an affinity-purified antibody directed against tryptophan hydroxylase (affinity-purified sheep anti-tryptophan hydroxylase polyclonal antibody, cat. # 96260-2505, 1:10,000; Biogenesis, Poole, UK) or using (medulla only) an antibody directed against tyrosine hydroxylase (rabbit anti-tyrosine hydroxylase polyclonal antibody, AB152, 1:4000; Chemicon, Chandlers Ford, Hampshire, UK), using methods described previously (Abrams et al., 2005; Hollis et al., 2005). Briefly, for double immunostaining of c-Fos and tryptophan hydroxylase, free-floating tissue was incubated in Iwaki 24-well tissue culture plates (Appleton Woods, Birmingham, UK), washed in plastic tubs using mesh wells (Netwell, 15 mm diameter, 500 μm mesh, cat. # 3478; Corning Costar, Sunderland, UK), and gently shaken on an orbital shaker throughout double immunostaining. Tissue was first washed in 0.05 M PBS for 15 min, then incubated in 1% hydrogen peroxide in PBS for 15 min, washed again for 15 min in PBS, pre-incubated in phosphate-buffered saline containing 0.3% Triton X-100 (PBST) for 15 min, and then incubated for 12–16 h with rabbit anti-c-Fos antiserum in PBST. Tissue was then washed twice, 15 min each time, in PBST followed by incubation with a biotinylated swine anti-rabbit IgG polyclonal antibody (E0353, 1:200; DAKO, Ely, UK) in PBST for 90 min. Tissue was again washed twice, 15 min each time, in PBST followed by incubation with an avidin–biotin complex (PK-6100, 1:200; Vector, Peterborough, UK) in PBST for 90 min. Finally, tissue was washed for 15 min in PBST, 15 min in PBS, and then incubated in chromogen (Vector SG; diluted as recommended by Vector) in PBS for 15 min. Tissue was immediately washed in PBS for 15 min, incubated in 1% hydrogen peroxide in PBS for 15 min, washed in PBS for 15 min followed by PBST for 15 min, then incubated with rabbit anti-tryptophan hydroxylase antibody in PBST for 12–16 h. All subsequent steps were identical to those described above for the immunohistochemical localization of c-Fos-immunoreactivity, except for the secondary antibody incubation and substrate reaction. Following the primary antibody incubation and washes as described above, sections were incubated with a biotinylated rabbit anti-sheep IgG polyclonal antibody (PK-6106, 1:200; Vector) in PBST for 90 min. Following incubation with avidin–biotin complex and subsequent washes as described above, sections were incubated in a solution containing 0.01% 3,3′-diaminobenzidine tetrahydrochloride (DAB) and 0.0015% hydrogen peroxide in PBS for 20 min, and then washed in PBST for 15 min and PBS for 15 min. Brain slices were mounted on clean glass slides, dehydrated and cleared with xylene, then coverslipped using mounting medium (DPX; R. A. Lamb, London, UK). The reaction product of the tryptophan hydroxylase immunostaining was a golden–brown color and localized to the cytoplasm while the reaction product of the c-Fos immunostaining was a blue–black color and localized to the nucleus. Identical methods were used for double immunostaining of c-Fos and tyrosine hydroxylase within the medulla except that following immunostaining for c-Fos and subsequent washing steps, sections were incubated with rabbit anti-tyrosine hydroxylase antibody, instead of the sheep anti-tryptophan hydroxylase antibody, in PBST for 12–16 h, and sections were incubated with a biotinylated swine anti-rabbit IgG polyclonal antibody (E0353, 1:200; DAKO, Ely, Cambridgeshire, UK) instead of the biotinylated rabbit anti-sheep IgG polyclonal antibody.

#### Cell counting, imaging, and figure preparation

One brain section of the medulla at the level of the AP (approximately −7.48 mm Bregma) was selected from each mouse and used for analysis of c-Fos expression within the area postrema (AP) and dorsolateral part (SolDL) of the nucleus of the solitary tract (nTS). Two brain sections selected from the DRC, at approximately −4.84 and −5.02 mm Bregma containing the DRC and DRI were selected from each mouse and used for cell counts; the mean cell counts for each brain region from these two sections were used for statistical analysis. Identification of rostrocaudal levels and subdivisions of the brainstem raphe complex was based on comparisons of the immunostained tissue with a standard stereotaxic mouse brain atlas (Paxinos and Franklin, 2001) and an atlas of tryptophan hydroxylase immunostaining in the mouse brain (Abrams et al., 2004). All analysis was performed in a blind manner with respect to treatment groups after randomization of slides. Cell counts of c-Fos-like-immunoreactive (c-Fos-ir) nuclei in the AP or nTS and the numbers of c-Fos-positive serotonergic neurons in the DRC and DRI were performed using a Leica DMLS microscope with a 40× objective (Leica Microsystems, Germany) by an investigator that was blind to the experimental treatment of each mouse. All photographic images were captured using a Leica DMLB microscope fitted with an Insight digital camera (Leica Microsystems) and SPOT image capture software v4.0.2 (Diagnostic Instruments, Sterling Heights, MI, USA). All graphs were made using SigmaPlot 8.0 software (Systat Software, London, UK) and all figures were designed and assembled in CorelDRAW 12.0 (Corel Corporation, Eden Prairie, MN, USA).

#### Measurement of l-tryptophan, 5-HT, and 5-HIAA concentrations

Brain microdissection combined with HPLC and electrochemical detection of l-tryptophan, 5-HT, and 5-HIAA, was based on a previously described procedure (Renner and Luine, 1984). Frozen brain tissue was sectioned using a cryostat and serial 300 μm sections were thaw-mounted onto clean glass microscope slides, rapidly re-frozen and stored at −80 °C until microdissection. Individual brain regions were microdissected at −10 °C using the Palkovits punch technique (Palkovits and Brownstein, 1982). Using a 1 mm i.d. stainless steel microdissecting needle specific regions of the ventromedial prefrontal cortex and hippocampus were microdissected as follows: the prelimbic cortex was sampled at 2.84 and 2.54 mm Bregma, the infralimbic cortex was sampled at 1.94 and 1.64 mm Bregma and the medial hippocampal CA1 region was sampled at −2.06 and −2.36 mm Bregma. All microdissections were done bilaterally and samples from the left and right side of each region were analyzed separately. Microdissected tissues in individual brain regions from each hemisphere from individual mice were expelled into separate tubes containing 60 μl acetate buffer (pH 5), and then stored at −80 °C until they were analyzed for tissue concentrations of l-tryptophan, 5-HT, and 5-HIAA. For analysis samples were thawed and centrifuged at 13,000 r.p.m. for 2 min; 50 μl of the supernatant from each sample was then placed in an ESA 542 autosampler (ESA Analytical, Ltd., Huntington, UK) maintained at 4 °C. Ten microliters of supernatant from each sample was then injected onto the chromatographic system.

Plasma samples were analyzed for total plasma concentrations of l-tryptophan. The assay method was based on a previously described procedure (Pussard et al., 1996). Plasma samples were thawed at 4 °C and 50 μl of each sample was used for analysis. Ten microliters of 10% sodium borohydride and 50 μl of 20% perchloric acid were added to each sample. Samples were vortexed and centrifuged at 13,000 r.p.m. for 3 min; the supernatant from each sample was then placed in an ESA 542 autosampler (ESA Analytical) maintained at 4 °C. A volume of 10 μl of supernatant from each sample was then injected onto the chromatographic system.

For HPLC analysis of indole concentrations, chromatographic separation was accomplished using an integrated precolumn/column system consisting of a guard cartridge (4.6×5 mm) attached to an Ultrasphere XL-ODS cartridge (4.6×70 mm; Beckman Coulter, Fullerton, CA, USA). The mobile phase consisted of 9.53 g/l KH_2_PO_4_, 200 mg/l 1-octanesulfonic acid, and 35 mg/l EDTA in 13% methanol; pH was adjusted to 3.4–3.5 using orthophosphoric acid. Electrochemical detection was accomplished using an ESA Coulochem II multi-electrode detector with an ESA 5021 conditioning cell and an ESA 5011 analytical cell with electrodes set at −0.10 and +0.55 V. The mean peak heights (pg/cm) of known concentrations of l-tryptophan, 5-HT, and 5-HIAA standards were determined from the peak heights of two chromatographs for each respective standard. Concentrations of l-tryptophan, 5-HT, and 5-HIAA in samples were determined based on peak heights measured using a computerized analysis system (EZChrom Elite for Windows, ver 2.8; Scientific Software, Inc., Pleasanton, CA, USA) while the analyst was blind to the nature of the treatment groups.

#### Forced swim test

Exposure to the forced swim test was performed 12 h and 36 h following s.c. challenge with whole heat-killed *M. vaccae* or vehicle in *M. vaccae*– or saline-preimmunized mice. A 12 h time point was chosen to coincide with the timing of *M. vaccae*–induced increases in 5-HT metabolism in the medial prefrontal cortex. We reasoned that if increased 5-HT metabolism reflects increased 5-HT release, and if 5-HT release in the medial prefrontal cortex alters emotional behavior, then we should observe effects of *M. vaccae* on behavior in the forced swim test at the 12 h time point. Mice were individually placed in a 4 l plastic beaker (height, 21 cm, diameter, 16 cm at the top) filled with water to a depth of 14 cm for 6 min; the water was maintained at a constant temperature of 23 °C. Behavior was scored for the final 5 min period of the test during which investigators were absent from the room. A digital video camera (JVC, GR-D70EK, JVC London, London, UK) was used to record behavior. Behavior was scored as the duration of time each mouse spent 1) swimming, 2) climbing or struggling, and 3) immobile. A mouse was scored as immobile when it stopped moving completely, except for minor movements of the tail. Antidepressant-like behavioral effects are related to a reduction in immobility time (Borsini, 1995). Behavioral analysis was conducted using the Noldus Observer software program (ver. 5.0; Noldus, Wageningen, The Netherlands). All behavior was scored by a single observer blind to the experimental treatment of individual mice.

### Statistics

All statistical analyses used Statistical Package for the Social Sciences (SPSS) version 11.5.0 (SPSS, Woking, UK), and all reported values are mean values and standard errors of the means (S.E.M.). Comparisons of two independent means were made using Student’s *t*-test. Comparisons among means in experimental designs with multiple between subjects factors were analyzed using multifactor analysis of variance (ANOVA) followed, when appropriate, by post hoc analysis using Fisher’s protected least significant difference (LSD) tests. For the cell count data each dependent variable measured (e.g. the number of c-Fos immunostained nuclei, the number of c-Fos/tryptophan hydroxylase double-immunostained cells) was analyzed using a single multifactor ANOVA with repeated measures analysis, using TREATMENT (e.g. *M vaccae* antigen; *M. vaccae*; OVA) or TIME as the between-subjects factors and REGION (e.g. each subdivision of the brainstem raphe complex analyzed) as the within-subjects factor for repeated measures analysis. For cell counts the left and right sides of the brain were not distinguished from each other so the cell count data represent the total of the left and right sides. Missing values in the cell count data were replaced by the method of Petersen (1985) prior to the multifactor ANOVA with repeated measures analysis but the original data were used for post hoc analysis and for representation of the data in figures. For analysis of neurochemical data, tissue concentrations of l-tryptophan, 5-HT and 5-HIAA were analyzed within each region with a single multifactor ANOVA with repeated measures using TREATMENT as the between-subjects factor and HEMISPHERE (e.g. left or right hemisphere of each microdissected forebrain region) as the within-subjects factor for repeated measures analysis. Missing values were not replaced for analysis of neurochemical data. When a TREATMENT effect or an interaction between TREATMENT and REGION was observed, the appropriate post hoc pair-wise comparisons were made using Fisher’s protected LSD tests. A two-tailed alpha level of 0.05 was used to determine statistical significance.

## Results

### *M. vaccae* and subsets of serotonergic neurons

#### Experiment 1

**Fig. 1:**
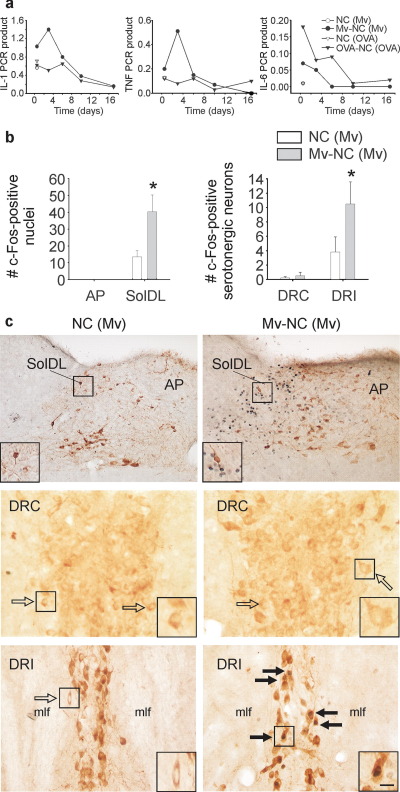
Influence of *M. vaccae* or its derivatives on pulmonary cytokine mRNA expression, c-Fos expression in the nTS and c-Fos expression in serotonergic neurons in the dorsal raphe nucleus (DR). (a) Graphs illustrate mean levels, relative to β-actin, of pulmonary IL-1β (IL-1), TNF-α (TNF), and IL-6 mRNA expression 12 h, and 3, 6, 10, and 17 days following i.t. injection of Mv-NC in *M. vaccae*–preimmunized mice (•) relative to cytokine mRNA expression following i.t. injection of vehicle in *M. vaccae*–preimmunized mice (○) (12 h time point only), as well as, for comparison, cytokine mRNA expression at the same time points following i.t. injection of OVA-NC in OVA/alum-preimmunized mice (▾) or vehicle in OVA/alum-preimmunized mice (▿) (12 h time point only). (b) Bar graphs illustrate the mean number (±S.E.M.) of c-Fos-ir nuclei in the AP, SolDL, DRC, and DRI in experiment 1. (c) Photographs illustrate nuclear c-Fos immunostaining (blue–black) 12 h following i.t. injection of *M. vaccae* in *M. vaccae*–preimmunized mice in the AP and SolDL of the nTS (top two photographs) and serotonergic neurons in the DRC (middle two photographs) and DRI parts (bottom two photographs) of the DR. Tyrosine hydroxylase immunostaining (brown) was used to aid in identification of neuroanatomical subdivisions of the nTS. Tryptophan hydroxylase immunostaining (brown) was used to identify serotonergic neurons in the DRC and DRI. (⇒) c-Fos-immunonegative serotonergic neurons, () c-Fos-immunopositive serotonergic neurons. Small black boxes in (c) indicate regions shown at higher magnification in insets. Scale bar=100 μm (c) top row; (c) middle and bottom rows, 25 μm; (c) insets, 12.5 μm. Abbreviations: IL-1, interleukin-1β; (Mv), preimmunization with s.c. injections of heat-killed *M. vaccae*; Mv-NC, i.t. challenge with sonicated heat-killed Mv-NC; NC, i.t. challenge with NC. * *P*≤0.05, compared with *M. vaccae*–preimmunized, vehicle-injected controls, Fisher’s protected LSD test. For interpretation of the references to color in this figure legend, the reader is referred to the Web version of this article.

We used a model of localized bronchopulmonary immune activation to investigate the effects of immunomodulation and subsequent peripheral immune activation on afferent vagal pathways and serotonergic systems. In order to induce a localized bronchopulmonary immune activation, sonicated, heat-killed *M. vaccae* was coupled to NC and was administered through the i.t. route to adult male BALB/c mice preimmunized with whole heat-killed *M. vaccae*. Control mice were also preimmunized with whole heat-killed *M. vaccae* but received i.t. injections of NC alone. Consistent with a Th1-biased bronchopulmonary immune response, *M. vaccae*–preimmunized mice challenged with i.t. Mv-NC had relatively higher pulmonary IL-1β and TNFα mRNA expression levels at 12 h compared with vehicle-treated controls and at 12 h and 3 days compared with OVA/alum-preimmunized mice challenged with i.t. OVA-NC (Fig. 1a). In contrast, consistent with a Th2-biased bronchopulmonary immune response, OVA/alum-preimmunized mice challenged with i.t. OVA-NC had relatively higher pulmonary IL-6 mRNA expression levels at 12 h compared with vehicle-treated controls and at 12 h, 3 days, and 6 days compared with *M. vaccae*–preimmunized mice challenged with i.t. Mv-NC (Fig. 1a). Consistent with a localized bronchopulmonary immune response (as opposed to a systemic immune response) in preimmunized mice challenged with Mv-NC, we found that plasma concentrations of IL-1β, TNF-α, and IL-6 were below the limits of detection at all time points studied (12 h, 3, 6, 10, and 17 days; limits of detection IL-1β, <7.8 pg/ml; TNF-α, <23.4 pg/ml; IL-6, <15.6 pg/ml).

To determine if i.t. administration of Mv-NC results in a pattern of brain signaling consistent with involvement of afferent vagal fibers, we quantified c-Fos-ir nuclei within the SolDL at the level of the AP (Fig. 1b, c). The SolDL is a principal target within the nTS of afferent vagal fibers originating in the extrathoracic trachea, intrathoracic trachea, main bronchi, and lungs (Kalia and Mesulam, 1980). The AP is a circumventricular organ which, due to the lack of a blood–brain barrier, is responsive to circulating cytokines in plasma (Maier et al., 1998). The rapid and intense induction of c-Fos protein in the CNS is an effective tool for detecting functional intracellular responses at single cell resolution among large populations of neurons (Sheng and Greenberg, 1990; Morgan and Curran, 1991). Bronchopulmonary immune activation with Mv-NC increased the number of c-Fos-ir nuclei within the SolDL 12 h following Mv-NC challenge, compared with vehicle-injected controls. In contrast there were no effects of Mv-NC challenge within the AP (Fig. 1b, c). In addition, there were no effects of treatment on c-Fos expression within the nTS at later time points studied (3, 6, 10, 17 day; data not shown).

Bronchopulmonary challenge with Mv-NC also increased c-Fos expression within specific subsets of serotonergic neurons. At 12 h following i.t. challenge with Mv-NC, Mv-NC-challenged mice had increased numbers of tryptophan hydroxylase-ir (serotonergic) neurons that also contained c-Fos immunoreactivity, compared with vehicle-treated control mice. Increases in the numbers of c-Fos-ir/tryptophan hydroxylase-ir neurons in Mv-NC-challenged mice were found in the distinct bilateral columns of serotonergic neurons located within the DRI (Fig. 1b, c; −4.8 to −5.0 mm Bregma; *M. vaccae* effect, *F*_(1,8)_=11.045; *P*<0.05). In contrast, in the DRC (Fig. 1b, c), i.t. challenge with Mv-NC had no effect on the number of serotonergic neurons containing c-Fos-ir nuclei. The treatment had no effect on c-Fos expression in DRI or DRC serotonergic neurons at any of the later time points studied (3, 6, 10, 17 days; data not shown).

### Impact of the type of immune response

#### Experiment 2

**Fig. 2:**
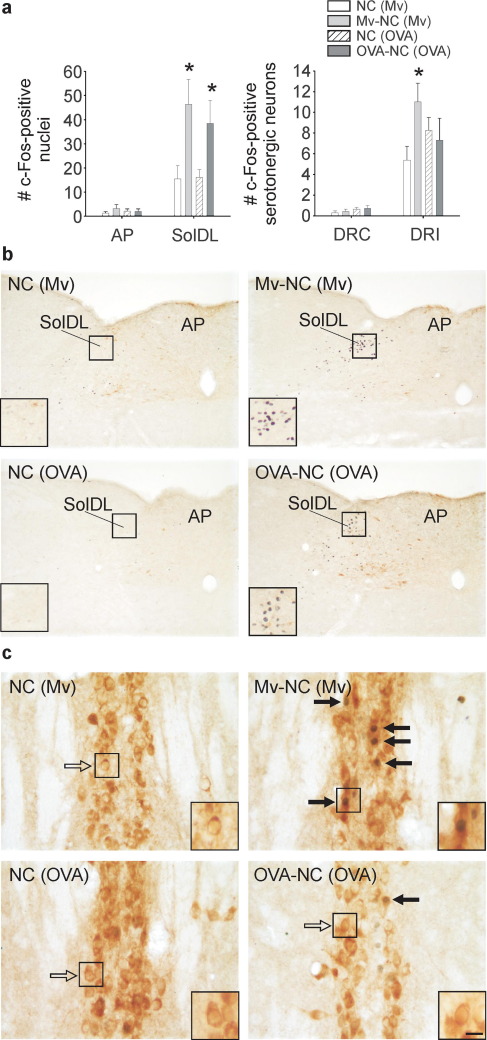
Both i.t. Mv-NC and i.t. OVA-NC induced c-Fos expression in the nTS but only Mv-NC increased c-Fos expression in DRI serotonergic neurons. Immunostained products are the same as in Fig. 1. (a) Bar graphs illustrate the mean number (±S.E.M.) of c-Fos-ir nuclei in the AP and SolDL (left) and c-Fos-ir/tryptophan hydroxylase-ir neurons in the DRC and DRI (right) in experiment 2. (b, c) Photomicrographs illustrate c-Fos responses to Mv-NC in *M. vaccae*–preimmunized mice or to OVA-NC in OVA/alum-preimmunized mice in the SolDL and AP (b) or the DRI (c). (⇒) c-Fos-immunonegative serotonergic neurons, () c-Fos-immunopositive serotonergic neurons. Small black boxes indicate regions shown at higher magnification in insets. Scale bar=100 μm b; b (insets), 50 μm; c, 25 μm; c (insets) 12.5 μm. Abbreviations: (OVA), preimmunization with s.c. injections of OVA/alum; OVA-NC, i.t. challenge with OVA-NC. For additional abbreviations, see Fig. 1 legend. * *P*≤0.05, compared with the appropriate *M. vaccae*– or OVA/alum-preimmunized, vehicle-injected control group, Fisher’s protected LSD test.

To determine if the effects of peripheral immune activation on serotonergic systems in the DRI were dependent on the type of peripheral immune response, we compared the effects of i.t. administration of Mv-NC in *M. vaccae*–preimmunized mice to the effects of i.t. administration of OVA-NC in OVA and alum-preimmunized mice. We chose to challenge mice that had been preimmunized with *M. vaccae*, or alternatively with OVA on alum, because these immunization schedules induce polarized immune responses. Mv-NC induces a Th1-biased immune response and promotes the development of Treg cells (Abou-Zeid et al., 1997; Zuany-Amorim et al., 2002), whereas OVA in alum induces a Th2-dominant immune response characterized by release of IL-4 (Gerhold et al., 2002). We found that challenge with either i.t. Mv-NC or i.t. OVA-NC increased c-Fos expression in the SolDL, but not in the AP, at the 12 h time point (Fig. 2a, b, *M. vaccae*×*region* interaction, *F*_(1,14)_=6.332; *P*<0.001; *OVA*×*region* interaction, *F*_(1,14)_=5.644; *P*<0.001), suggesting that both stimuli activated afferent vagal fibers arising from the bronchopulmonary system. In contrast, Mv-NC, but not OVA-NC, increased c-Fos expression in DRI serotonergic neurons (Fig. 2a, c; *M. vaccae*×*region* interaction, *F*_(1,11)_=12.622; *P*<0.01). Neither Mv-NC nor OVA-NC altered c-Fos expression in DRC serotonergic neurons (Fig. 2a).

### The role of afferent vagal pathways

#### Experiment 3

Previous studies have demonstrated that afferent fibers traveling within the vagus nerve are involved in relaying signals of peripheral immune activation to the CNS (Maier et al., 1998). To determine if pharmacological activation of bronchopulmonary afferent vagal fibers is sufficient to stimulate c-Fos expression in DRI serotonergic neurons, we activated afferent bronchopulmonary fibers by injecting the 5-HT_3_ receptor agonist, PBG, into the right atrium of the heart to stimulate cardiac and bronchopulmonary receptors. Injections of PBG into the right atrium resulted in components of the expected Bezold-Jarisch cardiopulmonary reflex (apnea, bradycardia, and hypotension) (Coleridge and Coleridge, 1984). Right atrial injections of PBG induced a reflex bradycardia (control, 590±15 beats per min; PBG, 328±32 beats per min). Although not analyzed there was a cessation of breathing lasting 2–8 s. Following PBG injection, there was evidence of increased afferent vagal signaling as judged by the number of c-Fos-ir nuclei in the SolDL measured 2 h after injection (see de Vries et al., 2005). The topographical distribution of c-Fos expression within the nTS was similar to that observed following i.t. administration of *M. vaccae*, with c-Fos expression almost entirely restricted to the SolDL region (de Vries et al., 2005), a major target of bronchopulmonary afferent vagal fibers (Kalia and Mesulam, 1980). In contrast, PBG had no effect on c-Fos expression in DRI or DRC serotonergic neurons (DRI, vehicle 4±2.3; PBG 3.4±0.9 neurons; DRC, vehicle 0.1±0.2, PBG 0±0 neurons).

#### Experiment 4

**Fig. 3:**
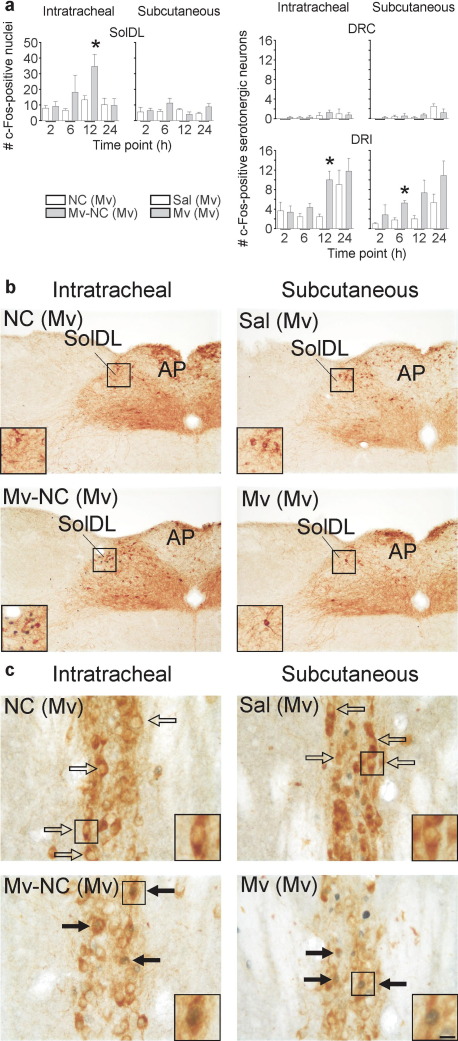
Activation of bronchopulmonary afferent vagal pathways was not necessary for the effects of Mv-NC on DRI serotonergic neurons. Immunostained products are the same as in Fig. 1. (a) Bar graphs illustrate the mean number (±S.E.M.) of c-Fos-ir nuclei in the AP and SolDL (left) and c-Fos-ir/tryptophan hydroxylase-ir neurons in the DRC and DRI (right) in experiment 4. (b, c) Photomicrographs illustrate c-Fos responses to i.t. Mv-NC or s.c. *M. vaccae* in *M. vaccae*–preimmunized mice in the SolDL and AP (b) or the DRI (c) at the 12 h time point. For abbreviations, see Fig. 1 legend. (⇒) c-Fos-immunonegative serotonergic neurons, () c-Fos-immunopositive serotonergic neurons. Scale bar=100 μm b; b (insets), 50 μm; c, 25 μm; c (insets), 12.5 μm. Abbreviations: Mv, s.c. challenge with heat-killed *M. vaccae*; Sal, s.c. challenge with saline vehicle. For additional abbreviations, see Fig. 1 legend. * *P*≤0.05, compared with the appropriate *M. vaccae*–preimmunized, vehicle-injected control group, Fisher’s protected LSD test.

To further characterize the time course of the effects of *M. vaccae* and to determine if a bronchopulmonary site of peripheral immune stimulation and, therefore, activation of specific afferent fibers is necessary for the effects of immune stimulation on DRI serotonergic neurons, we investigated the effects of both i.t. and s.c. injections of Mv-NC in preimmunized mice on c-Fos expression in the SolDL and in DRI serotonergic neurons, measured 2, 6, 12, and 24 h following injection. For s.c. injections, we used whole heat-killed *M. vaccae*. We found that i.t. injection of Mv-NC, but not s.c. injection of *M. vaccae* in preimmunized mice, increased c-Fos expression in the SolDL (Fig. 3a, b; i.t. Mv-NC effect, *F*_(1,35)_=5.934, *P*<0.05). However, both i.t. injections of Mv-NC and s.c. injections of *M. vaccae* increased c-Fos expression in DRI (but not DRC) serotonergic neurons, although the effects of s.c. injections were observed earlier than the effects of i.t. injections (6 h versus 12 h) (Fig. 3a, c; i.t. Mv-NC effect, *F*_(1,23)_=7.180; *P*<0.05; s.c. *M. vaccae* effect, *F*_(1,23)_=9.859; *P*<0.01). The effects of s.c. injections of *M. vaccae* also approached statistical significance at the 12 h time point (*P*=0.059). Because both i.t. injections of Mv-NC and s.c. injections of *M. vaccae* increased c-Fos expression in DRI serotonergic neurons, we used s.c. *M. vaccae* injections in all subsequent studies.

### *M. vaccae* and limbic serotonergic systems

#### Experiment 5

**Fig. 4:**
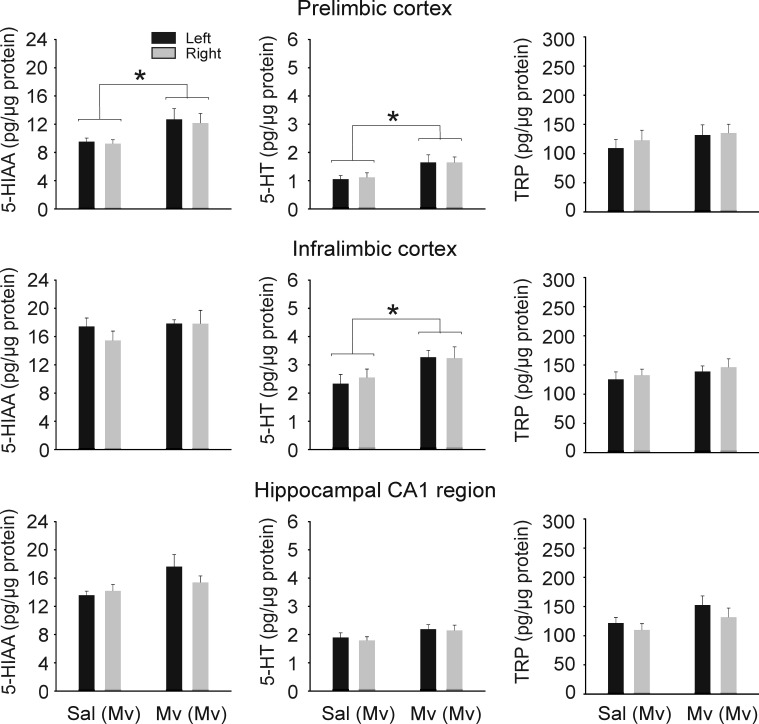
S.c. challenge with whole heat-killed *M. vaccae* in *M. vaccae*–preimmunized mice increased 5-HT and 5-HT metabolite concentrations in the medial prefrontal cortex. Graphs illustrate 5-HIAA, 5-HT, and l-tryptophan concentrations in the left and right hemispheres of each brain region 12 h following s.c. injections of vehicle or whole heat-killed *M. vaccae* in *M. vaccae*–preimmunized mice (*n*=8–9). Abbreviations: Mv, s.c. challenge with heat-killed *M. vaccae*, Sal, s.c. challenge with saline vehicle; TRP, l-tryptophan. For additional abbreviations, see Fig. 1 legend. * *P*≤0.05, compared with *M. vaccae*–preimmunized, vehicle-injected controls based on multifactor ANOVA with repeated measures analysis within each brain region.

In order to determine if the effects of *M. vaccae* on c-Fos induction in DRI serotonergic neurons reflect functional activation of serotonergic pathways, we measured 5-HT metabolism in forebrain target regions of DRI serotonergic neurons 12 h following s.c. challenge with *M. vaccae*. Retrograde tracing studies have demonstrated that large numbers of serotonergic neurons within the DRI give rise to projections to specific limbocortical structures involved in mood regulation including the prefrontal cortex (Porrino and Goldman-Rakic, 1982; Van Bockstaele et al., 1993) and hippocampus (Azmitia, 1981). Consequently, we microdissected selected forebrain targets of DRI serotonergic neurons including the medial prefrontal cortex (prelimbic cortex, infralimbic cortex), and the medial CA1 region of the dorsal hippocampus. S.c. injection of *M. vaccae* in preimmunized mice increased 5-HT and 5-HIAA concentrations in some brain regions but not others (Fig. 4; prelimbic cortex: *M. vaccae* effect on 5-HT, *F*_(1,16)_=5.275; *P*=0.035; *M. vaccae* effect on 5-HIAA, *F*_(1,15)_=4.932; *P*=0.042; infralimbic cortex: *M. vaccae* effect on 5-HT, *F*_(1,16)_=5.352; *P*=0.034). The effects of s.c. injection of *M. vaccae* on tissue concentrations of 5-HIAA within the medial CA1 region of the dorsal hippocampus, a major forebrain target of the DRI (Azmitia, 1981), approached statistical significance (Fig. 4; *P*=0.076). Tissue concentrations of 5-HT, 5-HIAA and tryptophan did not differ between left and right hemispheres. There were no effects of treatment on total plasma concentrations of l-tryptophan, the amino acid precursor of 5-HT (vehicle, 12.8±1.5 μg/ml, *M. vaccae* 14.2±2.1 μg/ml; *t*_16_=−0.542, *P*=0.595) or tissue concentrations of l-tryptophan (Fig. 4).

### Behavioral effects of *M. vaccae* in the forced swim test

#### Experiment 6

**Fig. 5:**
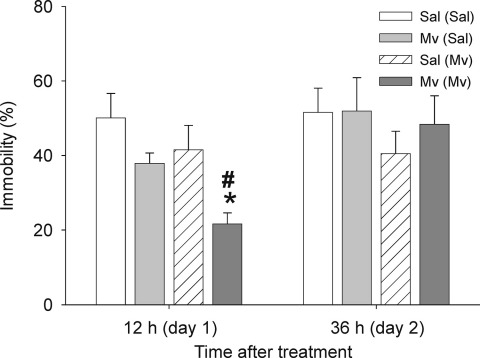
S.c. challenge with whole heat-killed *M. vaccae* in *M. vaccae*–preimmunized mice decreased immobility in the forced swim test measured 12 h following challenge. Abbreviations: (Sal), s.c. preimmunization with saline vehicle. For additional abbreviations, see Fig. 1 legend. ^#^ *P*<0.05 compared with *M. vaccae*–preimmunized, saline-challenged control group. * *P*<0.01 compared with saline-preimmunized, *M. vaccae*–challenged control group.

Mice were evaluated in the forced swim test 12 h post-treatment, when effects of *M. vaccae* on 5-HT and 5-HIAA concentrations in the medial prefrontal cortex were observed, and again 36 h post-treatment. We found that preimmunized mice challenged with *M. vaccae* displayed decreased immobility in the forced swim test 12 h following treatment compared with preimmunized mice that were challenged with vehicle (Fig. 5). In contrast, there were no effects of either *M. vaccae*– or vehicle-challenge in mice that had not been preimmunized with *M. vaccae*. The behavioral effects seen in preimmunized mice were no longer apparent when mice were re-tested 36 h following *M. vaccae* challenge (Fig. 5).

## Discussion

We have identified what appears to be an anatomically and functionally distinct population of mesolimbocortical serotonergic neurons that are uniquely responsive to peripheral immune activation. The discrete location of the immune responsive serotonergic neurons suggests that they may be Type II serotonergic neurons, serotonergic neurons that have unique sensory response patterns and unique behavioral correlates and appear to be restricted to the DRI region (Rasmussen et al., 1984). The effects of immune activation on serotonergic neurons within the DRI were observed 6–12 h following treatment and appear to be due to the type of peripheral immune response as they were observed following Th1- and Treg-biased immune activation but not following Th2-biased immune activation. These effects were temporally associated with increases in 5-HT metabolism in the medial prefrontal cortex, a forebrain target of DRI serotonergic neurons, and with altered behavioral responses in the forced swim test, consistent with previous studies demonstrating that peripheral immune activation can alter stress-related emotional behavior. Activation of DRI serotonergic neurons may be a general mechanism through which peripheral immune activation influences physiological, behavioral, or cognitive processes.

Data from the present study are consistent with a localized immune activation within the bronchopulmonary system without significant systemic involvement 12 h following i.t. administration of Mv-NC. Plasma concentrations of several cytokines including IL-1β, TNF-α, and IL-6 were undetectable 12 h following treatment. In addition, in marked contrast to systemic immune activation (Maier et al., 1998), i.t. administration of Mv-NC had no effects on c-Fos expression within the AP, a circumventricular organ that is responsive to circulating cytokines. Nevertheless, it is possible that c-Fos expression was present in the AP at earlier time points, and the lack of c-Fos expression is not conclusive evidence for a lack of neuronal activation (Kovacs, 1998). Bronchopulmonary immune activation following i.t. administration of Mv-NC increased c-Fos expression within the dorsolateral part (SolDL) of the nTS, consistent with anatomical studies in cats in which the dorsolateral part (together with the ventrolateral part) of the nTS at the level of the AP receives the greatest input from afferent vagal fibers arising from the intrathoracic trachea and upper right lobe of the lung (Kalia and Mesulam, 1980), and consistent with our findings that bronchopulmonary, but not s.c. immune activation, increased c-Fos expression in the SolDL. Finally, intra-atrial injections of the 5-HT_3_ receptor agonist PBG, which are thought to selectively activate cardiopulmonary afferent vagal fibers (Coleridge and Coleridge, 1984), increased c-Fos expression in the SolDL and the adjacent gelatinous part of the nTS at the level of the AP, but not other subdivisions of the nTS (see de Vries et al., 2005). These data suggest that i.t. administration of Mv-NC induced a localized bronchopulmonary immune response that activated bronchopulmonary afferent vagal fibers.

Increases in c-Fos expression in the nTS and in DRI serotonergic neurons occurred 12 h following challenge with Mv-NC, consistent with previous studies describing effects of peripheral immune activation on extracellular concentrations of 5-HT in limbic brain regions (including the prefrontal cortex and hippocampus, which are innervated by DRI serotonergic neurons (Azmitia, 1981; Porrino and Goldman-Rakic, 1982)), elevating extracellular concentrations of 5-HT up to 8 h following immune challenge (Dunn, 1992; Lavicky and Dunn, 1995; Linthorst et al., 1996). Typically, the maximal level of c-Fos protein expression occurs between 1 and 3 h following an acute challenge; c-Fos then gradually disappears from the cell nucleus by 4–6 h after challenge (Kovacs, 1998). Therefore, the detection of c-Fos protein 12 h following immune challenge suggests that Mv-NC-induced signaling within the nTS and DRI serotonergic neurons was secondary to a stimulus with a delayed onset, between 6 and 11 h following i.t. challenge with Mv-NC. Presumably this delay reflects the need for the immune response to develop and suggests that the increased c-Fos expression is an effect of the immune response to *M. vaccae*, not a direct pharmacological effect of *M. vaccae* components.

Bronchopulmonary or s.c. immune activation with Mv-NC or *M. vaccae*, respectively, increased c-Fos expression within serotonergic neurons in the DRI but not in the adjacent DRC. Based on the restricted anatomical location of these effects, peripheral immune activation by *M. vaccae* may have selective actions on Type II serotonergic neurons that have been described previously based on studies *in vivo* (Rasmussen et al., 1984; Sakai and Crochet, 2001). Indeed, Type II serotonergic neurons were only found at the caudal interface of the dorsal and median raphe nuclei, between the mlf (Rasmussen et al., 1984). As the neuronal activity of Type II serotonergic neurons is not correlated with behavioral arousal or motor activity (Rasmussen et al., 1984), a selective effect of *M. vaccae* on Type II serotonergic neurons could explain the dissociation between serotonergic neurotransmission and behavioral arousal that has been observed following peripheral immune stimulation (Linthorst et al., 1995).

The effects of *M. vaccae* on DRI, but not DRC, serotonergic neurons, support the hypothesis that there is physiological and functional diversity among midbrain serotonergic neurons, consistent with previous studies (Kocsis et al., 2006; Rasmussen et al., 1984; Sakai and Crochet, 2001). The subpopulation of serotonergic neurons we have identified that is responsive to immune stimulation is not the same as the subpopulation of serotonergic neurons that responds to multiple anxiety-related stimuli, including multiple anxiogenic drugs such as the adenosine receptor antagonist caffeine, the 5-HT_2A/2C_ receptor agonist m-chlorophenylpiperazine (mCPP), and the partial inverse agonist at the benzodiazepine site of the GABA_A_ receptor, N-methyl-beta-carboline-3-carboxamide (FG-7142) (Abrams et al., 2005), the anxiety-related neuropeptide urocortin 2 (Staub et al., 2005), uncontrollable stress (Grahn et al., 1999), and social defeat (Gardner et al., 2005). Anxiety-related stimuli consistently activate serotonergic neurons within the DRC but, with the exception of a small effect of caffeine, not the DRI (Abrams et al., 2005; Gardner et al., 2005), while treatment with *M. vaccae* or LPS (Hollis et al., 2006) activates serotonergic neurons within the DRI, but not the DRC. The neural basis of this anatomical specificity is not clear, however, subdivisions of the anxiety-related bed nucleus of the stria terminalis appear to selectively innervate the dorsomedial part of the mid-rostrocaudal DR and DRC, but not the DRI region (Dong et al., 2001). The restricted distribution of these afferents could account for the selective effects of aversive or anxiety-related stimuli on DRC serotonergic neurons. Unfortunately, afferents to the DRI have not been studied and identification of candidate neural pathways that may selectively regulate DRI neurons will require further studies. Nevertheless, our findings are consistent with previous studies showing that Type I serotonergic neurons, such as those distributed throughout the dorsal raphe and median raphe nucleus, and Type II serotonergic neurons, which have only been described between the mlf in the caudal part of the midbrain raphe complex (i.e. the DRI), respond differently to phasic auditory or visual stimulation (Rasmussen et al., 1984). Type I serotonergic neurons respond to phasic auditory or visual stimulation with short-latency, short-duration excitations, while Type II serotonergic neurons in the DRI respond with short-latency, long-duration inhibitions (Rasmussen et al., 1984). It remains to be determined if differential responses of Type I and Type II serotonergic neurons to sensory stimulation are due to differences in intrinsic properties of the neurons or differences in afferent regulatory mechanisms.

Although DRC and DRI serotonergic neurons are in close proximity to each other, selective activation of DRC or DRI serotonergic neurons may have different behavioral outcomes. DRC serotonergic neurons are thought to be involved in behavioral despair (escape deficits in a shuttle box paradigm) and facilitation of anxiety-related behaviors in a model of learned helplessness (Maier and Watkins, 2005). In contrast, our data suggest that peripheral immune activation may have antidepressant-like behavioral effects via actions on DRI serotonergic neurons. The mechanisms underlying the proposed alternative behavioral responses following activation of DRC or DRI serotonergic neurons are not clear. However, serotonergic neurons within the DRC project strongly to anxiety-related brain regions including the amygdala and the bed nucleus of the stria terminalis (Lowry et al., 2005). In contrast, DRI serotonergic neurons appear to project strongly to regions of the prefrontal cortex (including the frontal pole, dorsolateral prefrontal cortex, and medial orbitofrontal cortex), and anterior cingulate cortex in primates (Porrino and Goldman-Rakic, 1982), areas that are generally acknowledged to be important for regulation of affective and cognitive processes (Vertes, 2006). The medial prefrontal cortex has been implicated in coping responses and even the inhibition of DRC serotonergic neurons (Amat et al., 2005). The possibility that DRI and DRC serotonergic neurons may interact via long-loop feedback mechanisms is particularly intriguing and, if such a mechanism is present in humans, could have implications for understanding the mechanisms underlying the high incidence of comorbid anxiety and depression (Pollack, 2005; Gulley and Nemeroff, 1993).

The present studies suggest that the type of immune activation in the periphery is an important determinant of the effects on DRI serotonergic neurons. The effects were observed following challenge with Mv-NC in *M. vaccae*–preimmunized mice but not following challenge with OVA in OVA/alum preimmunized mice which induces a qualitatively different, Th2-dominant immune response (Gerhold et al., 2002). It will be important to determine, in future studies, the unique qualitative or quantitative properties of immune activation that are important for its effects on DRI serotonergic systems. The recent finding that i.p. injections of high concentrations of lipopolysaccharide (LPS; a component of gram-negative bacteria) increase c-Fos expression in DRI serotonergic neurons but not several other subpopulations of serotonergic neurons studied in mice, including the DRC (Hollis et al., 2006), suggests that the effects of peripheral immune activation on DRI serotonergic neurons are not unique to *M. vaccae* but may be dependent on shared qualitative properties of the immune response.

The neural mechanisms through which peripheral immune activation increased c-Fos expression in DRI serotonergic neurons are not clear. However, the weight of the evidence suggests that these effects were not mediated by activation of the vagus nerve, a major afferent signaling pathway from the periphery to the CNS (Maier et al., 1998). Evidence from the present studies suggests that activation of bronchopulmonary afferent vagal fibers, at least the subpopulations activated by PBG or by an OVA-induced Th2-dominant immune response, is not sufficient for activation of DRI serotonergic neurons. Meanwhile, s.c. administration of *M. vaccae* had no effect on c-Fos expression in the region of the nTS innervated by bronchopulmonary afferent vagal fibers but increased c-Fos expression in DRI serotonergic neurons, suggesting that activation of bronchopulmonary afferent vagal fibers is not necessary for activation of DRI serotonergic neurons. There is no evidence for a direct innervation of the DRI by afferent vagal fibers, suggesting that the effects of peripheral immune activation on these serotonergic neurons were via multisynaptic pathways or other indirect mechanisms. Potential mechanisms include signaling via afferent fibers in the spinal cord (Coleridge and Coleridge, 1984), via cytokines or prostaglandins acting at the level of the cerebrovasculature or circumventricular organs (Maier et al., 1998), via convergence of both neural and humoral signaling at the level of the CNS (Dantzer et al., 2000), or via indirect mechanisms such as changes in physical parameters, including body temperature (Hori and Harada, 1976; Bratincsak and Palkovits, 2004) or pH (Richerson, 2004), that can alter the activity of serotonergic systems.

Although it is clear from the present studies that a localized bronchopulmonary immune activation can activate afferent vagal pathways, it is unlikely that these pathways account for the effects of *M. vaccae* (following either i.t. or s.c. administration) on DRI serotonergic neurons. The potential for convergence at the level of the spinal cord of somatosensory and viscerosensory signals of peripheral immune activation deserves further study. It is likely that signals of visceral immune activation are relayed to the brain via afferent fibers traveling within the sympathetic nerve bundles (innervating the spinal cord), in addition to those traveling within the vagus nerve bundles (innervating the dorsomedial medulla). Free sensory nerve endings are observed within the epithelial lining of the trachea and lungs, often associated with specialized 5-HT-containing neuroendocrine cells, either neuroepithelial endocrine cells or neuroepithelial bodies (Adriaensen and Scheuermann, 1993; Cutz and Jackson, 1999). The cell bodies of primary sensory afferents innervating the epithelial lining of the upper airways and lungs are mainly located in the nodose (60%) and jugular ganglia (20%) of the vagus nerve but approximately 20% are located in the T1–T5 dorsal root ganglia, mainly at the T2–T3 levels, and a few are located in the stellate and superior cervical ganglia (Dalsgaard and Lundberg, 1984). Although the function of these “sympathetic afferents” is largely unknown (Coleridge and Coleridge, 1984), T2–T3 spinal neurons within lamina I and deeper laminae do respond to irritation of the lower airways and receive convergent noxious somatic input (Hummel et al., 1997; Qin et al., 2006). Furthermore, functional studies support a role for sympathetic afferents in regulation of the neuronal activity of brainstem serotonergic neurons. The vast majority of raphespinal neurons (i.e. raphe neurons with descending projections to the spinal cord, identified by antidromic activation from the T2–T5 segments) in the raphe obscurus (ROb) and approximately 65% of raphe spinal neurons within the raphe magnus (RMg) respond to stimulation of thoracic sympathetic afferents (stimulation of the left stellate ganglion) (Blair and Evans, 1991). Although the study by Blair and Evans (1991) focused on neurons in the ROb and RMg with descending projections, stimulation of sympathetic afferents is also likely to affect neurons within the RMg with ascending projections. Ascending projections from the RMg innervate the medial reticular formation and strongly innervate the area immediately lateral and ventral to the mlf in the region of the DRI (Bobillier et al., 1976). Future studies will be required to determine if the effects of i.t. or s.c. *M. vaccae* on DRI serotonergic neurons are dependent on these spinal afferent pathways.

The effects of *M. vaccae* on DRI serotonergic neurons were temporally associated with increases in 5-HT metabolism in the prefrontal cortex, a forebrain target of serotonergic neurons in the DRI region (Porrino and Goldman-Rakic, 1982; Van Bockstaele et al., 1993). Although the functional consequences of increased 5-HT metabolism in the medial prefrontal cortex are uncertain, previous studies in rodents suggest that mesolimbocortical serotonergic systems play an important role in coping with stressors (Graeff et al., 1996). Based on these findings and previous studies in humans demonstrating that administration of *M. vaccae* improves quality of life scores (O’Brien et al., 2004), we predicted that *M. vaccae* would have antidepressant-like behavioral effects in mice. S.c. administration of *M. vaccae* in *M. vaccae*–preimmunized mice resulted in decreased immobility in the forced swim test (an antidepressant-like effect), when measured 12 h, but not 36 h, after treatment.

The precise relationships among immune activation-induced c-Fos expression in DRI serotonergic neurons, 5-HT metabolism in the medial prefrontal cortex, and behavior in the forced swim test will need to be addressed in future studies. Stimulus-induced increases in c-Fos expression typically represent activation from 1 to 3 h earlier (Kovacs, 1998). The effects of s.c. injection of *M. vaccae* on c-Fos expression in DRI serotonergic neurons were significant at the 6 h time point and nearly significant at the 12 h time point. It is therefore likely that the effects of *M. vaccae* on 5-HT metabolism in the prefrontal cortex and behavior in the forced swim test would also occur at earlier time points (i.e. 3–9 h following s.c. injection of *M. vaccae*). Further studies will be required to resolve the full time course of the effects of *M. vaccae* on 5-HT metabolism in the prefrontal cortex and on emotional behavior.

A recent study has also described antidepressant-like behavioral effects in the forced swim test following administration of LPS in mice (Renault and Aubert, 2006). Interestingly, mice treated with LPS responded with anti-depressant-like behavioral effects when tested 90 min following treatment but responded with depressant-like behavioral effects when tested a second time 24 h after treatment. The mechanisms underlying the opposed, time-dependent effects of LPS on behavior in the forced swim test are not certain, but the antidepressant-like behavioral effects were observed at a time soon after maximal levels of mesolimbocortical serotonergic neurotransmission are observed (30–60 min following i.p. administration of LPS) (Linthorst et al., 1996), consistent with the time course of activation of DRI serotonergic neurons following LPS administration (4 h following i.p. LPS administration) (Hollis et al., 2006). Other studies have reported no effects (Deak et al., 2005) (2 h after i.p. injection in rats), or depressant-like effects (Dunn and Swiergiel, 2005) (90 min after i.p. injection in mice) of LPS in the forced swim test. Studies using other proinflammatory compounds and other behavioral tests have yielded equivocal results (reviewed by Dunn et al., 2005). The reasons underlying the differences in findings among studies of the effects of LPS and *M. vaccae* in the forced swim test are unclear, although there are several methodological differences. For example, in addition to the qualitative differences in the immune response elicited by LPS and *M. vaccae*, and the time courses of behavioral testing, our study and the study by Renault and Aubert (2006) used lower water temperatures (23–24 °C), compared with the studies by Deak and colleagues (2005; 25 °C) and Dunn and Swiergiel (2005; 30 °C). Future studies should be able to clarify the mechanisms underlying the differential behavioral effects of peripheral immune activation in these studies.

Although there remain many unanswered questions, we hypothesize that under some conditions, acute peripheral immune activation activates DRI serotonergic neurons and has antidepressant-like behavioral effects, but that following chronic peripheral immune activation, afferent mechanisms signaling to the DRI, or the signaling mechanisms of DRI serotonergic neurons themselves, downregulate, leading to a decrease in mesolimbocortical serotonergic neurotransmission and a depressed state. Although, as we have demonstrated in this study, i.t. administration of *M. vaccae* initially increases pro-inflammatory cytokine (IL-1β and TNF-α) mRNA expression locally within the bronchopulmonary system, this effect dissipates after a few days. Long-term effects of *M. vaccae* may be due to its immunoregulatory properties, for example its effects on Treg cells leading to production of anti-inflammatory cytokines including IL-10 and TGF-β (Zuany-Amorim et al., 2002).

We expected, but did not detect, increases in 5-HT metabolism in the hippocampus following administration of *M. vaccae*, although an effect approached statistical significance. The serotonergic innervation of the hippocampus is topographically organized (Lowry, 2002) and it is possible that the effects of immune activation on serotonergic systems in the hippocampus are limited to a subset of serotonergic fibers innervating specific domains within the hippocampus. Based on the temporal relationships among c-Fos expression in the DRI, 5-HT metabolism in the prefrontal cortex, and behavioral responses in the forced swim test it will be important to determine if DRI serotonergic neurons, via projections to forebrain limbic sites, play an important role in regulation of stress-related emotional behavior.

## Conclusions

Administration of either whole or ultrasonically disrupted preparations of heat-killed *M. vaccae* increased c-Fos expression in a subpopulation of serotonergic neurons within the DRI, increased 5-HT metabolism in the prefrontal cortex, and altered emotional behavior. Mesolimbocortical serotonergic systems, particularly those in the medial prefrontal cortex where we observed effects of *M. vaccae* on serotonergic metabolism, are thought to play an important role in regulation of coping responses and behavioral responses to uncontrollable stress (Graeff et al., 1996; Amat et al., 2005). Consequently, dysregulation of DRI serotonergic systems may contribute to the dysregulation of coping mechanisms in some stress-related neuropsychiatric disorders, including major depression. The effects of immune activation were dependent on the type of immune activation, suggesting that modulation of the balance of immune signaling in the periphery will have important consequences for brain serotonergic function. Identification of DRI 5-HT neurons as uniquely responsive to peripheral immune activation provides a novel hypothetical framework for investigating the relationships among immune activation, serotonergic systems, and mental health.
